# Effects of *Kalimeris indica* (L.) Sch Bip on colitis-associated colorectal cancer

**DOI:** 10.3389/fphar.2022.1119091

**Published:** 2023-01-09

**Authors:** Mo-Fei Wang, Hao Li, Jian Cui, Yu-Han Chen, Yong Cui

**Affiliations:** ^1^ The Department of General Surgery, The Affiliated Hospital of Inner Mongolia University for the Nationalities, Tongliao, Inner Mongolia Autonomous Region, China; ^2^ School of Traditional Chinese Materia Medica, Shenyang Pharmaceutical University, Shenyang, China; ^3^ School of Medical Device, Shenyang Pharmaceutical University, Shenyang, China

**Keywords:** colitis associated cancer, microRNA, miR-31-5p, *Kalimeris indica* (L.) Sch Bip, colorectal cancer

## Abstract

*Kalimeris indica* (L.) Sch Bip (*K. indica*) is a plant of the genus *Kalimeris* in Asteraceae, and its whole herb can be used as medicine for the treatment of intestinal inflammatory diseases. But the mechanism is not clear. Therefore, this study was designed to explore the mechanism of *K. indica* (KI) in colitis-associated colorectal cancer. The expression levels of miR-31-5p and proinflammatory factors were detected using THP-1 and Caco2 cells *in vitro*. KI could rescue the upregulation of miR-31-5p induced by IL-6 and TNF-α in Caco2 and THP-1 cells. In LPS-stimulated PMA-differentiated THP-1 cells, KI restored miR-31-5p expression by downregulating the expression of IL-6 and TNF-α. C57BL/6 mice were used to construct CAC model through the induction of azoxymethane/dextran sulfate sodium. The successfully established CAC mice were treated with water extract of KI through intragastric administration for 5 weeks. The result showed that KI could significantly reduce the atypical hyperplasia in colon tissue, and inhibit the expression of proinflammatory factors such as IL-6, TNF, IL-11, IL-7, etc. At the same time, KI could restore the level of miR-31-5p in mice, and therefore the downstream LATS2 to inhibit the development of CAC. These above results indicate that KI is a potentially effective herb medicine to prevent CAC.

## 1 Introduction

Colorectal cancer (CRC) is one of the most common malignant tumors of the digestive system in clinic, and its incidence rate and mortality rate are among the top three in all cancers (Bray et al., 2018). According to the etiological classification, CRC can be divided into hereditary, sporadic, and colitis-associated colorectal cancer (CAC). The incidence of colorectal cancer in patients with inflammatory bowel disease such as ulcerative colitis is higher than that in the general population (Dugum et al., 2017; Limidi and Farraye, 2018; Samadder et al., 2019; Olén et al., 2020). The epidemiological investigation found that the occurrence of various tumors is related to inflammation, and chronic inflammation of intestinal mucosa is an important risk factor for CRC. No clear adenoma is formed during the development of CAC, and its pathogenesis was mainly attributed to the repeated cycle of the intestinal epithelial cell (IECs) injury and healing process (Rajamäki et al., 2021). Therefore, the pathogenic factors of CAC include the degree and duration of chronic inflammation, genetic susceptibility, symbiotic microbiota, etc. Sporadic CRC often develops from adenomatous polyps to cancer, and its pathogenesis is an adenoma-cancer process, while the pathogenesis of CAC goes through the sequence evolution of inflammation-atypical hyperplasia-cancer (Baker K T et al., 2018). Because the lesion boundary of CAC is not clear, it is difficult to diagnose it by the ordinary electronic endoscopy (Buchner and Lichtenstein, 2016; Hata et al., 2016; Baker A M et al., 2018; Mark-Christensen et al., 2018; Gui et al., 2020).

With the application of genome and epigenome methods and gene-modified mouse models, the molecular mechanism of CAC has been greatly promoted, but the exact molecular mechanism of the transformation from inflammation to cancer is still unclear. Chronic inflammation can cause intestinal mucosa damage, mutation, and epigenetic changes of cell proliferation-related genes, and finally lead to atypical hyperplasia and oncogenesis (Romano et al., 2016; Kameyama et al., 2018; Fantini and Guadagni, 2021). There are two main forms of oncogenesis in colitis, including villous process oncogenesis and flat mucosa oncogenesis. CAC is usually asymptomatic in the early stage. With the progress of the disease, the focus gradually increases, and the local and systemic effects on the body gradually increase, resulting in a series of symptoms, such as changes in stool characteristics and defecation habits, abdominal masses, acute and chronic intestinal obstruction, intestinal perforation, peritonitis, and other manifestations. Due to the different nature, location, and course of the tumor, its clinical manifestations differ to some extent (Okayasu et al., 1996; Liu et al., 2021). Chemotherapy and surgery are the main treatment for CAC clinically. Fluorouracil and molecular targeted drugs are clinical drugs but with many adverse reactions (Fiala et al., 2019; Ginghină et al., 2021). At the same time, there is a possibility of complications in the above treatments, and long-term medication will also reduce the compliance of patients. With the increasing number of CAC patients in China, traditional Chinese medicine (TCM) plays a very important role in the treatment of CAC. Many research experiments on the treatment of CAC with TCMs have been carried out, and many prescriptions have begun to be used in clinical practice.

*Kalimeris indica* (L.) Sch Bip (*K. indica*) is a plant of the genus *Kalimeris* in Asteraceae, and its whole herb can be used as a folk medicine (Wang G K et al., 2019). *K. indica* (KI) is rich in plant resources and widely distributed (Wang L et al., 2019). It is mainly grown in low-altitude humid environments such as roadsides and hillsides. Because of its large resource stock and low development cost, *K. indica* has the basis for large-scale development and application. As a common folk medicine, it has a long medicinal history (Maoa et al., 2015) with its application recorded firstly in the *Bencao Shiyi* (741 AD). Chinese Pharmacopoeia records that *K. indica* has the effect of regulating the flow of vital energy, digesting food, and clearing away dampness and heat, and can be used to treat epigastric pain, dysentery, enteritis, urinary tract infection, etc., (Wang et al., 2010; Wang et al., 2015; Wang et al., 2017). *K. indica* mainly contains phenolic acids, flavonoids, alkaloids, phytosterols, triterpenoids, volatile oils, polysaccharides, et al. (Lin et al., 2006; Ji et al., 2014; Ueda et al., 2014; Wang et al., 2017; Huang, et al., 2020). Pharmacological studies showed that it has anti-inflammatory and analgesic, anti-tumor, anti-virus, blood coagulation improving, and other pharmacological effects. At present, *K. indica* is not only a folk medicine but also the main component of various Chinese commercial medicines. The therapeutic effect of *K. indica* on intestinal inflammatory diseases has been practiced, but its pharmacological effect on tumors needs to be clarified. In this study, the therapeutic effect of *K. indica* on CAC mice was evaluated for the first time, and its related mechanism of alleviating colitis-associated colorectal cancer was explored.

## 2 Materials and methods

### 2.1 Cells culture

Thp-1 and Caco2 cells were purchased from ATCC. The two cell lines were cultured in the medium of MEM supplemented with 10% fetal bovine serum (v/v) and 100U/mL penicillin and streptomycin (Solarbio Life Sciences Co., Ltd.) at 5% CO_2_ at 37°C. Cells were subcultured using trypsin when reaching 80% confluency.

### 2.2 Animals and treatment

C57BL/6 mice were obtained from Liaoning Changsheng Biotechnology Co., Ltd. All experiments were performed in Liaoning Changsheng Biotechnology Co., Ltd. The study protocol was approved by the Ethics Committee for Animal Experiments of Liaoning Changsheng Biotechnology Co., Ltd. Azoxymethane (AOM) was prepared at the concentration of 10 μg/μl. Dextran sulfate sodium (DSS) was prepared to 3.0% DSS in dH_2_O when needed. The experimental procedure was according to the method with modification (Chen et al., 2019). Briefly, mice were divided into four groups including the control group, AOM/DSS group, and KI groups (15 and 30 mg/kg daily). In AOM/DSS group, and KI groups, AOM of 12.5 mg/kg was administrated, i. p., respectively. After 7 days, 2.5% DSS (v/v) was prepared to drink for 7 consecutive days, followed by 14 days of regular water and normal feed, which constitutes the first 21-day cycle. Then the 21-day cycle was repeated twice. KI extract was orally administrated at dosages of 15 and 30 mg/kg from the fourth to the eighth week. Mice were sacrificed at the end of the tenth week. Serum and colon tissues were obtained and stored at −80°C for analysis.

### 2.3 Preparation of *K. indica* extract

*K. indica* was obtained from Anguo Herb Material Co., Ltd. (Anguo, China). And the herb sample was deposited with voucher specimen No. 19KI0516. The dried herb of *K. indica* (2.5 kg) was soaked in water for 60 min, and then refluxed with water (10 L) for 3 times, 1.5 h each time. After combining the KI extraction, 0.43 kg of the extract was obtained under vacuum.

### 2.4 Western blot assay

The whole cell lysis suspension was prepared and centrifuged at 4°C at a rate of 15,000 G for 15 min. Take the centrifuged supernatant and boil it for 5 min. The obtained samples were separated by SDS/PAGE gel containing 10% acrylamide. Add primary antibodies recognizing P-p65, p65, IκB, GPADH and LATS2 and incubate at room temperature for 120 min. After washing, continue to add secondary antibody, incubate again at room temperature for 60 min, and detect the results through chemiluminescence.

### 2.5 Bio-plex assay and ELISA assay

Bio-Rad Bio-Plex Assay specific kits were used to test the concentrations of G-CSF, and MIP-1β concentrations in serum following the manufacturer’s instructions. Contents of interleukins including IL-6, IL-11, IL-17A, IL-22, and IL-23 in serum were examined with ELISA method following the manufacturer’s instructions.

### 2.6 RT-PCR analysis

Total RNA was isolated by using the RNeasy mini kit (Qiagen) and used in RT and PCR amplification and the reverse transcription was used with the Master Mix kit (Takara, Shiga, Japan) following the standard protocol to make cDNA. For miRNA quantification: Bulge-loop miRNA qRT-PCR Primer Sets specific for miR-31-5p was designed by RiboBio. Mouse U6 was used as an endogenous control to normalize for total RNA loaded. RT-PCR and subsequent calculations were performed by the Step One Plus Real-time PCR system, which detected the signal emitted from fluorogenic probes during PCR.

### 2.7 Statistics

The data were analyzed by the statistics software of SPSS and all the data were expressed as means and standard deviations (Mean ± SD). The significance of data differences compared between groups by ANOVA. *p* < 0.05 means the difference is statistically significant.

## 3 Results

### 3.1 Effects of KI on miR-31-5p levels in cells treated with IL-6/TNF-α

**FIGURE 1:**
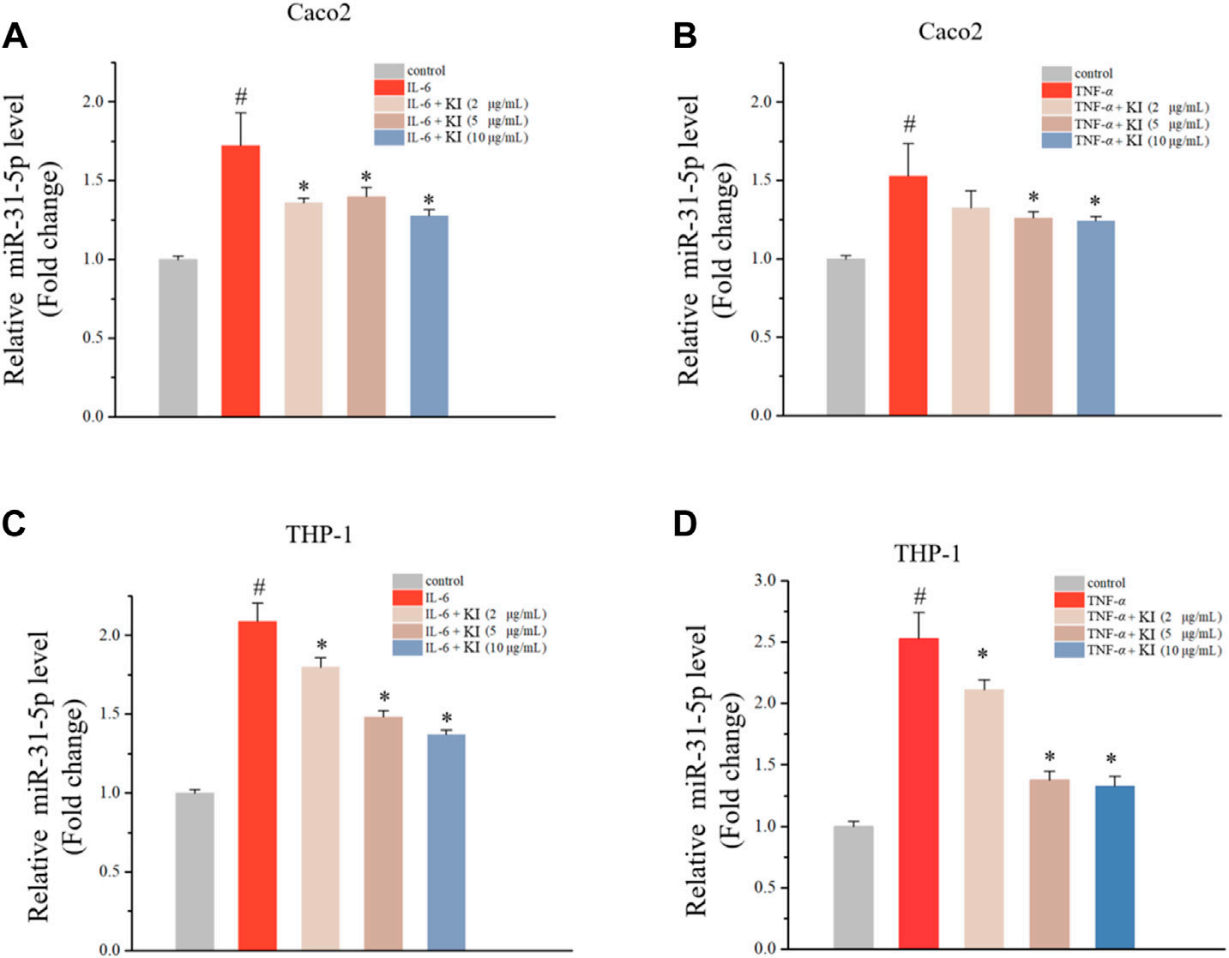
Levels of miR-31-5p in Caco2 and THP-1 cells treated with IL-6 or TNF-α. **(A–B)** RT-qPCR results of miR-31-5p in Caco2 cells stimulated by IL-6 **(A)** or TNF-α **(B)** for 48 h **(C–D)** RT-qPCR results of miR-31-5p in THP-1 cells stimulated by IL-6 **(A)** or TNF-α **(B)** for 48 h. Values are means ± SD from three separate experiments. ^#^ *p* < 0.05 compared with the control group; ^*^ *p* < 0.05 compared with the IL-6 **(A)** and **(C)** or TNF-α **(B)** and **(D)** groups.

MiR-31 is ectopically expressed in CAC by modulating Wnt signaling pathway (Liu et al., 2017; Song et al., 2020; Bocchetti et al., 2021). Therefore, the level of miR-31-5p was assessed in THP-1 and Caco2 cells stimulated by IL-6 and TNF-α indicating that IL-6 and TNF-α could elevate miR-31-5p levels (Figure 1). Subsequently, the effect of KI on the IL-6/TNF-α-driven miR-31-5p upregulation was examined. As a result, KI (5 and 10 μM) potently reversed the overexpression of miR-31-5p both in THP-1 and Caco2 cells stimulated by IL-6 and TNF-α (Figure 1). All these results implied KI might be able to prevent CAC through anti-inflammatory effects.

### 3.2 Effect of KI on NF-κB signaling

**FIGURE 2:**
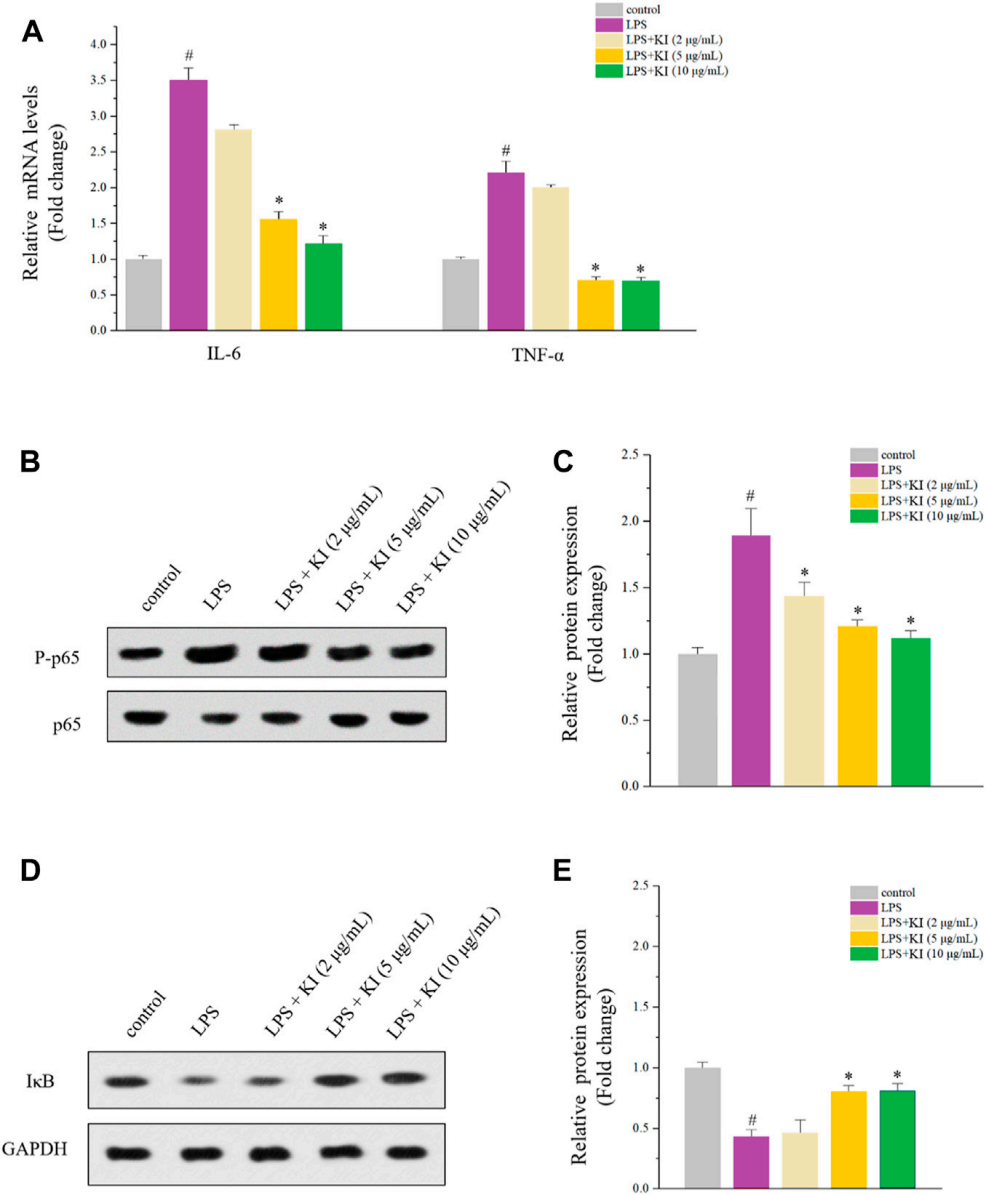
Effects of KI on LPS-stimulated THP-1 cells. **(A)** RT-qPCR results of IL-6 and TNF-α. PMA was used to differentiate THP-1 cells for 72 h and then the fresh PMA-free medium was used before the 24-h treatment of LPS or various concentrations of KI. Values are means ± SD from three separate experiments. ^#^ *p* < 0.05 compared with the control group; ^*^ *p* < 0.05 compared with LPS group. **(B)** Western blot results of P-p65/p65 of cells described in **(A)**. **(C)** Quantitative results of **(B)**. **(D)** Western blot results of P-p65/p65 of cells described in **(A)**. **(E)** Quantitative results of **(D)**. For **(D)** and **(E)**, values are means ± SD from three separate experiments. ^#^ *p* < 0.05 compared with the control group; ^*^ *p* < 0.05 compared with LPS group.

The anti-inflammatory activity of KI was evaluated, and PMA-differentiated THP-1 macrophage model was used. After the treatment of LPS, levels of IL-6 and TNF-α were increased while KI (5 and 10 μM) significantly reversed the overexpression of both TNF-α and IL-1β (Figure 2A). Similarly, KI (5 and 10 μM) also attenuated P-p65 expression and inhibited the degradation of IκB as indicated by the western blot experiment (Figures 2B–E). These data suggested that KI could restore miR-31-5p levels by inhibiting TNF-α and IL-6 expression in the NF-κB-dependent pathway.

### 3.3 KI on CAC induced by AOM/DSS

**FIGURE 3:**
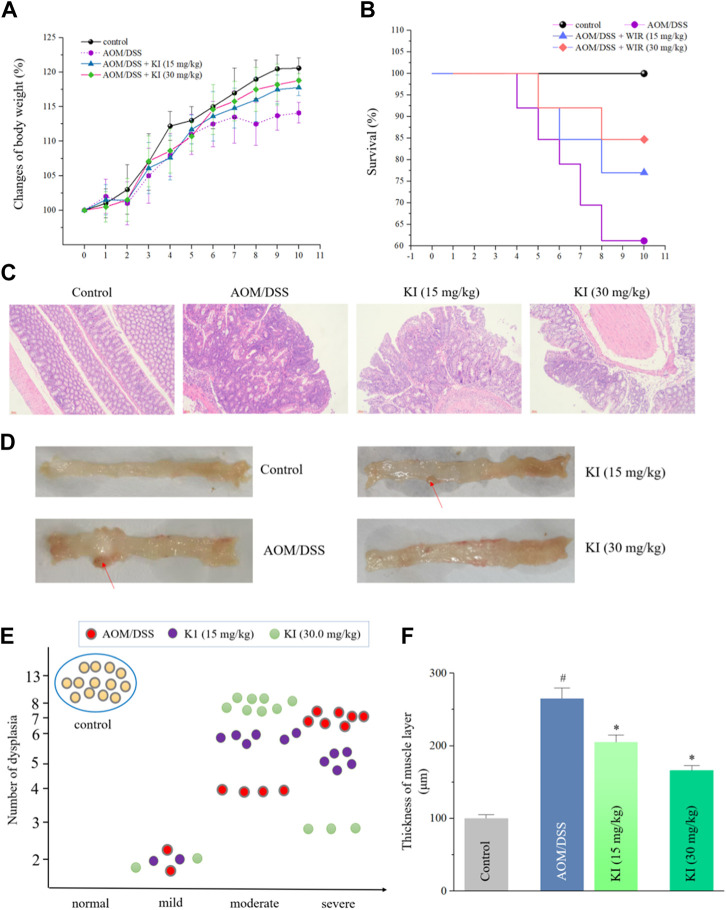
*In vivo* effects of KI on AOM/DSS-induced CAC. **(A)** Mean body weight (percentage) for each group (n = 13). Values are means ± SD from three separate experiments. **(B)** The survival rate for each group. **(C)** Histological changes for each group (× 200). **(D)** Representative macroscopic features of colon tissues for each group. Red arrows for the dysplasia. **(E)** Degrees of dysplasia event number (total = 13) for each group. **(F)** Thickness of muscle layer for each group. Values are means ± SD from three separate experiments. ^#^ *p* < 0.05 compared with the control group; ^*^ *p* < 0.05 compared with AOM/DSS group.

The AOM/DSS-induced CAC model was established to evaluate the *in vivo* preventive effect of KI (Zhang et al., 2021). There was no evident disparity in the mean body weight among each group except for the AOM/DSS group, indicating the toxic-free property of KI (Figure 3A). After treatment of KI (15 and 30 mg/kg) for five weeks, the CAC-induced mortality was decreased (survival rate: 84.6% for 30.0 mg/kg and 76.9% for 15 mg/kg in KI groups compared with 61.5% in AOM/DSS group) (Figure 3B). The AOM/DSS induced severe dysplasia and even tumors in the intestinal tissues after 10 weeks. KI (30.0 mg/kg) elicited an evident preventive effect on the intestine resulting in a reduction in the extent of intestinal injury and dysplasia (Figures 3C–E). Furthermore, increased thickness of the muscle layer was observed in the AOM/DSS group, which was to a large extent attenuated by the treatment of KI (30.0 mg/kg) (Figure 3F). All these preventive effect of KI (15.0 mg/kg) was much less significant than that of the KI (30.0 mg/kg) group (Figures 3C–F).

### 3.4 KI inhibited the expression of inflammatory cytokine in AOM/DSS-treated mice

**FIGURE 4:**
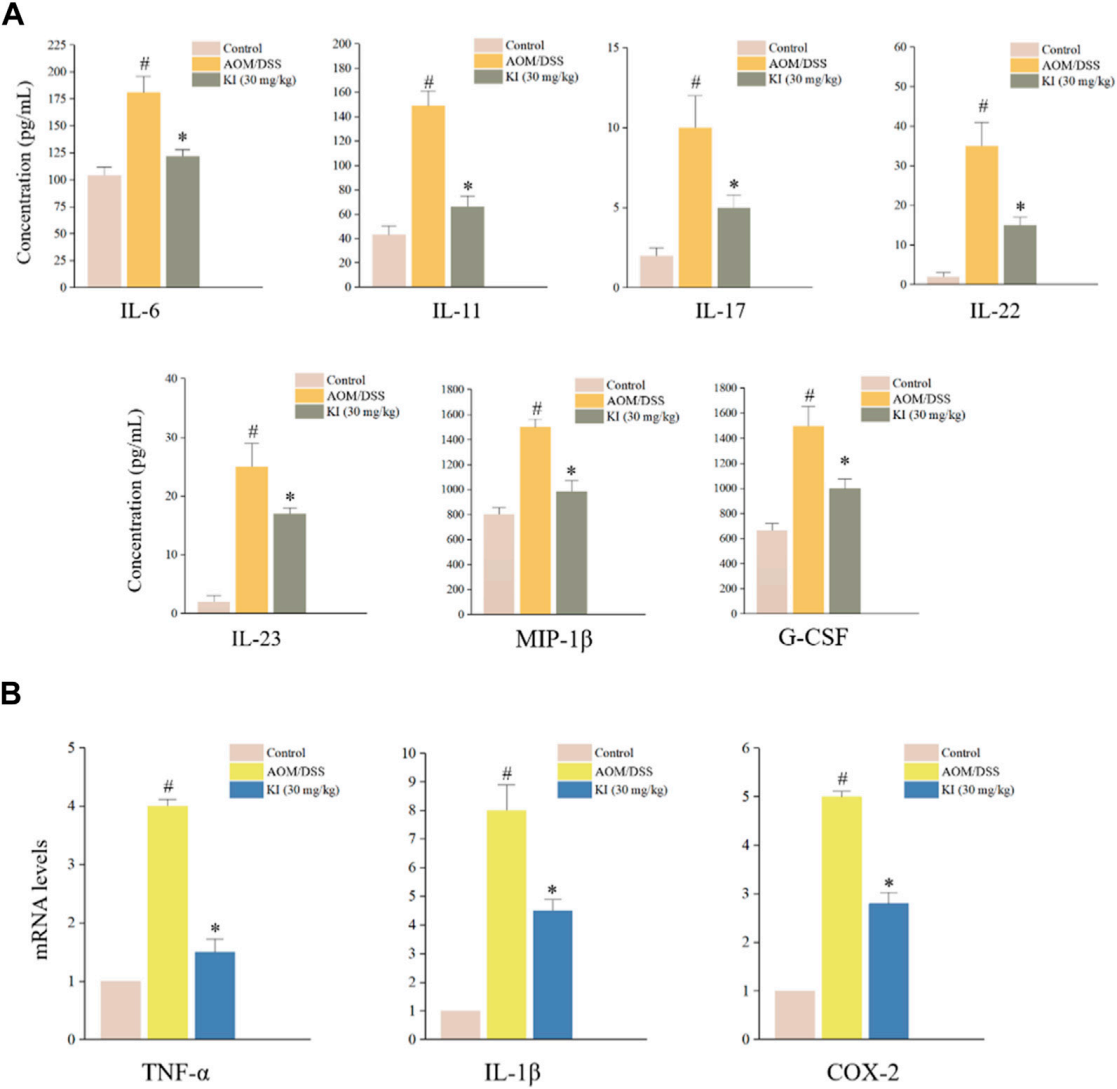
*In vivo* levels of inflammatory cytokines in CAC mice. **(A)** Results of Bio-Plex Assay or ELISA for indicated gene serum levels in mice at the 10th week (n = 6). **(B)** Relative levels of TNF-α, IL-1β, and COX-2 levels in mouse colons at 10th weeks (n = 6) assessed by RT-qPCR. Values are means ± SD from three separate experiments. ^#^ *p* < 0.05 compared with the control group; ^*^ *p* < 0.05 compared with AOM/DSS group.

Due to the result that KI at the dose of 30.0 mg/kg showed a more potent therapeutic effect in mice, we, therefore, tested the profile of *in vivo* inflammatory cytokines in the serum of KI (30.0 mg/kg) group. As a result, AOM/DSS increased the level of IL-6, IL-11, IL-17, IL-22, IL-23, MIP-1β, and G-CSF in serum, which was partially reversed by the KI (30.0 mg/kg) treatment (Figure 4A). In the colon tissue, mRNA expression of TNF-α, IL-1β, and COX-2 was elevated in the AOM/DSS group and KI group. KI lowered the overexpression of these genes in the colon tissue compared with the AOM/DSS group (Figure 4B).

### 3.5 KI decreases miR-31-5p expression and restores LATS2 level in colon tissues

**FIGURE 5:**
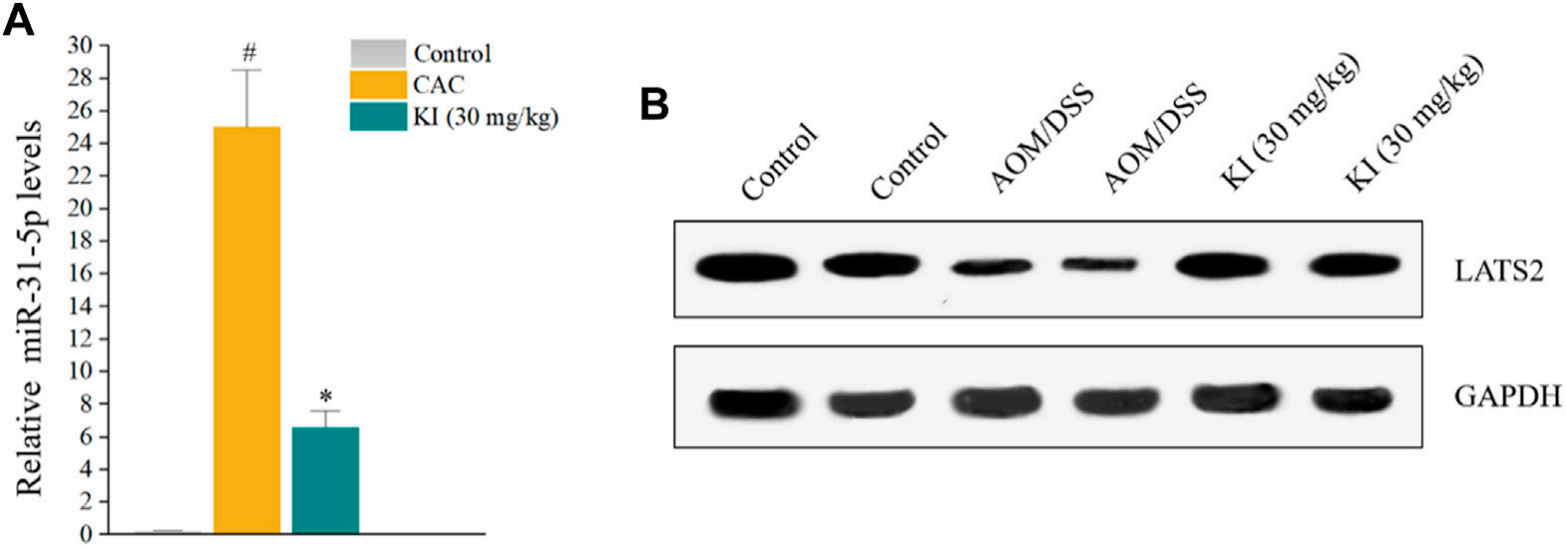
Effects of KI on miR-31-5p expression in CAC mice. **(A)** Rt-qPCR results of miR-31-5p in colon tissue at 10th week. Values are means ± SD from three separate experiments. ^#^ *p* < 0.05 compared with the control group; ^*^ *p* < 0.05 compared with AOM/DSS group. **(B)** Representative immunoblots of LATS2.

The *in vivo* level of miR-31-5p was examined to reveal if KI prevented CAC through a miR-31-5p-related mechanism as implied by *in vitro* experiments (Figure 1). The result indicated that miR-31-5p was overexpressed in the mice of AOM/DSS group compared with that of the control group and KI (30.0 mg/kg) significantly rescued the ectopic miR-31-5p overexpression caused by AOM/DSS (Figure 5A), suggesting miR-31-5p was involved in the KI-elicited preventive effect on CAC. To further confirm this notion, a western blot experiment was performed to assess the downstream LATS2 gene level of miR-31-5p. The protein level of LATS2 was attenuated in the AOM/DSS group due to the up-regulation of miR-31-5p and KI (30.0 mg/kg) partially restored the level of LATS2 of CAC mice (Figure 5B).

## 4 Discussion

MicroRNA, a kind of endogenous small RNAs with a length of about 20–24 nucleotides, can construct a regulatory network to modulate the expression of multiple genes. Through this complex network, miRNA with abnormal expression is always related to the high occurrence of diseases (Jin et al., 2018; Kang, 2018; Pheiffer et al., 2018). MiRNAs play an important role in the pathogenesis of CAC (Jaslin et al., 2020; Bocchetti et al., 2021), and this study explored the role of KI in regulating the miR-31-5p to prevent the development of CAC.

The results showed that the expression level of miR-31-5p was significantly up-regulated in CAC model mice. In addition, it was reported that mice with miR-31 knocked out would suffer from more serious CAC than wild-type mice (Liu et al., 2017). Based on these results, it can be concluded that the expression level of miR-31-5p will contribute to the occurrence and development of CAC in mice. Therefore, it can be considered that maintaining the stable and normal expression of miR-31-5p in mice plays a crucial role in preventing the occurrence of CAC. This experiment also investigated the mechanism of miR-31-5p upregulation in CAC and the results showed that proinflammatory cytokines IL-6 and TNF-α could enhance the expression of miR-31-5p. IL-6 and TNF-α in immune cells were overexpressed during CAC, and excessive IL-6 and TNF-α will stimulate the increase of expression level of miR-31-5p in intestinal epithelial cells. LATS2 is a downstream gene of miR-31-5p in the Wnt signaling pathway in intestinal epithelial cells. The decrease of LATS2 caused by the upregulation of miR-31-5p will lead to the blockage of YAP phosphorylation, the decrease in P-YAP entering the cytoplasm, and the relative increase of YAP in the nucleus, which will ultimately inhibit YAP repairing the damaged intestinal tissue cells in a short period of time during intestinal inflammation. Although the rapid increase of YAP can immediately start the repair of damaged intestinal tissue, there are still other risks of repeated damage and excessive repair because YAP also has a carcinogenic effect, increasing the risk of cancer. The possible pathway is that YAP accumulated in the nucleus will form YAP/TAZ complex and further trigger the transcription process of many downstream oncogenes. However, KI significantly reduced the level of YAP in CAC mice, suggesting that it could prevent the occurrence and development of CAC by regulating the cumulative amount of YAP, at least partially by restoring the normal level of miR-31-5p.

## Data Availability

The original contributions presented in the study are included in the article/supplementary material, further inquiries can be directed to the corresponding author.
